# HPA Axis Gene Expression and DNA Methylation Profiles in Rats Exposed to Early Life Stress, Adult Voluntary Ethanol Drinking and Single Housing

**DOI:** 10.3389/fnmol.2015.00090

**Published:** 2016-01-26

**Authors:** Aniruddha Todkar, Linnea Granholm, Mujtaba Aljumah, Kent W. Nilsson, Erika Comasco, Ingrid Nylander

**Affiliations:** ^1^Department of Neuroscience, Uppsala UniversityUppsala, Sweden; ^2^Department of Pharmaceutical Bioscience, Uppsala UniversityUppsala, Sweden; ^3^Centre for Clinical Research, Västerås Central Hospital, Uppsala UniversityUppsala, Sweden

**Keywords:** DNA methylation, early life stress, ethanol, gene expression, housing, hypothalamus, pituitary gland, rat

## Abstract

The neurobiological basis of early life stress (ELS) impact on vulnerability to alcohol use disorder is not fully understood. The effect of ELS, adult ethanol consumption and single housing, on expression of stress and DNA methylation regulatory genes as well as blood corticosterone levels was investigated in the hypothalamus and pituitary of adult out-bred Wistar rats subjected to different rearing conditions. A prolonged maternal separation (MS) of 360 min (MS360) was used to study the effect of ELS, and a short MS of 15 min (MS15) was used as a control. Voluntary ethanol drinking was assessed using a two-bottle free choice paradigm to simulate human episodic drinking. The effects of single housing and ethanol were assessed in conventional animal facility rearing (AFR) conditions. Single housing in adulthood was associated with lower *Crhr1* and higher *Pomc* expression in the pituitary, whereas ethanol drinking was associated with higher expression of *Crh* in the hypothalamus and *Crhr1* in the pituitary, accompanied by lower corticosterone levels. As compared to controls with similar early life handling, rats exposed to ELS displayed lower expression of *Pomc* in the hypothalamus, and higher *Dnmt1* expression in the pituitary. Voluntary ethanol drinking resulted in lower *Fkbp5* expression in the pituitary and higher *Crh* expression in the hypothalamus, independently of rearing conditions. In rats exposed to ELS, water and ethanol drinking was associated with higher and lower corticosterone levels, respectively. The use of conventionally reared rats as control group yielded more significant results than the use of rats exposed to short MS. Positive correlations, restricted to the hypothalamus and ELS group, were observed between the expression of the hypothalamus-pituitary-adrenal receptor and the methylation-related genes. Promoter DNA methylation and expression of respective genes did not correlate suggesting that other loci are involved in transcriptional regulation. Concluding, single housing is a confounding factor to be considered in voluntary ethanol drinking paradigms. ELS and ethanol drinking in adulthood exert independent effects on hypothalamic and pituitary related genes, however, in a manner dependent on the control group used.

## Introduction

**FIGURE 1:**
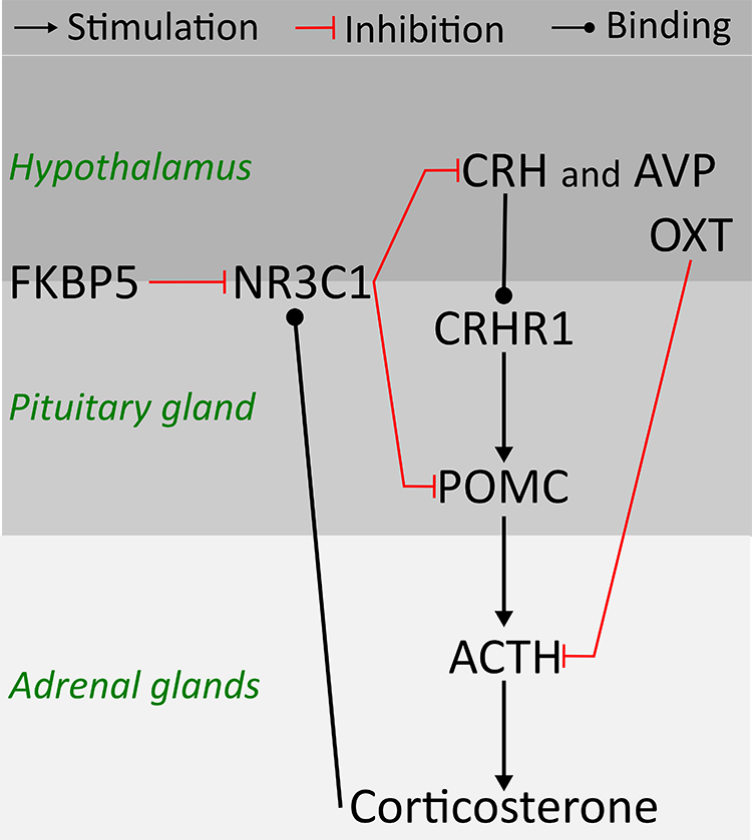
**Schematic representation of hypothalamus-pituitary-adrenal (HPA) axis physiology and relationship amongst candidate genes in rodents**.

Clinical studies point to early life stress (ELS) as a risk factor for psychiatric disorders, including alcohol use disorder (AUD) (Enoch, 2011). Preclinical studies provide evidence of increased risk for high ethanol consumption later in life as a consequence of ELS (Becker et al., 2011; Nylander and Roman, 2013). Environmentally induced changes in the stress response system and brain plasticity have been suggested to mediate long-term effects of ELS, however, previous findings are not consistent (Pryce and Feldon, 2003; Harrison and Baune, 2014; Maccari et al., 2014). The main system regulating the stress response is the hypothalamus-pituitary-adrenal (HPA) axis, a complex molecular pathway including feedback regulatory interactions between the hypothalamus, the pituitary gland, and the adrenal glands (**Figure 1**) (Tsigos and Chrousos, 2002). Corticotropin releasing hormone (CRH) and arginine vasopressin (AVP) released from the hypothalamus are essential regulators of the HPA axis. Through synergistic positive reinforcing interaction between CRH and AVP, and CRH actions on the CRH receptor (CRHR1), CRH and AVP stimulate the secretion of adrenocorticotropin hormone (ACTH) from the pituitary (Tsigos and Chrousos, 2002). ACTH is cleaved from proopiomelanocortin (POMC) (Smith and Funder, 1988), and stimulates the adrenal glands to secrete corticosterone, the principal glucocorticoid in rodents. Corticosterone binds to the glucocorticoid receptor (NR3C1) in the brain and inhibits the CRH release through an inhibitory feedback loop (Brunton, 2013). Moreover, the FK506 Binding Protein 5 (FKBP5) by changing the conformation of the receptor complex can reduce the sensitivity of NR3C1 to corticosterone and the efficiency of the negative feedback on the HPA axis (Binder, 2009). In addition to CRH and AVP, oxytocin (OXT) moderates the release of ACTH and corticosterone by either altering sensitivity to the feedback mechanism or by decreasing CRH release (Petersson et al., 1999).

DNA methylation can mediate the crosstalk between genome and environmental cues such as ELS into stable changes of the expression of key-genes of the HPA axis (Szyf, 2013; Maccari et al., 2014). Human as well as experimental studies suggest that ethanol is capable of producing global epigenetic modifications (Contet, 2012). Two important regulators of the DNA methylation machinery are the methyl CpG binding protein 2 (MECP2) and DNA (Cytosine-5)-methyltransferase 1 (DNMT1). MECP2 preferentially binds to methylated DNA and, through the recruitment of other proteins, represses gene expression, but also participates in the maintenance of DNA methylation during replication and reparation by recruiting DNMT1 which transfers methyl groups to hemi-methylated DNA (Moore et al., 2013). The mechanisms forming the relationship between ELS and ethanol consumption behavior in adulthood may involve long-term changes in epigenetic regulation of the HPA system but these mechanisms are largely unknown.

The present study used a rodent model to elucidate how ELS and adult voluntary ethanol drinking affect gene expression, DNA methylation, and corticosterone levels. Also, the effect of using two different but commonly used controls in rodent models of ELS on the outcome was also assessed. The focus was on a set of genes involved in stress response and DNA methylation, based on previous molecular and pharmacological findings (Gehlert et al., 2007; Murgatroyd et al., 2009; Klengel et al., 2013; Wu et al., 2014). The aim was to provide in-depth understanding of the biological mechanisms mediating the impact of stress and ethanol drinking in the hypothalamus and pituitary, which form the up-stream component of the stress system. The specific aims were to examine expression and DNA promoter methylation of genes regulating the HPA axis as well as corticosterone levels, and evaluate: (i) the effect of single housing and voluntary ethanol drinking in adult rats subjected to conventional early rearing conditions; (ii) the long-term effect of ELS alone and in combination with voluntary ethanol drinking; (iii) whether rearing condition and voluntary ethanol drinking affect correlation patterns in gene expression, corticosterone levels and ethanol drinking; and (iv) the results observed when conventionally rearing relative to short maternal separation (MS; 15 min) was used as control condition for prolonged MS (360 min).

## Materials and Methods

### Animals

Time mated Wistar dams (*n* = 25; RccHan:WI, Harlan, Europe) arrived at gestation day 15. After birth [postnatal day (PND) 0] the pups were sexed and cross-fostered to avoid the use of biological littermates in the same experimental groups. Each litter contained 10 pups, 6 males and 4 females, and the litters were randomly assigned to the different experimental groups. Only males were used in the present study. The study was approved by the Uppsala Animal Ethical Committee (C32/11) and followed the guidelines of the Swedish Legislation on Animal Experimentation (Animal Welfare Act SFS1998:56) and the European Communities Council Directive (86/609/EEC).

### Early Life Rearing Conditions

**FIGURE 2:**
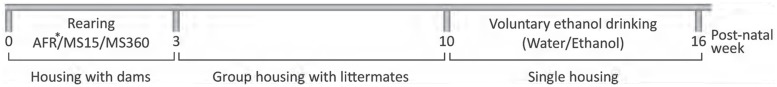
**Experimental outline.** ^∗^One additional group of AFR rats continued to be group-housed during adulthood to assess the effect of housing. AFR, animal facility rearing; MS, Maternal separation (15/360 min).

Pups from the same litters were randomized and subjected to three parallel groups; either MS (15/360 min) or conventional animal facility rearing (AFR) conditions. A rodent MS model was used to simulate ELS during the first three postnatal weeks (**Figure 2**). Based on previous studies, prolonged daily MS (360 min; MS360) was used to simulate ELS and short MS (15 min; MS15) was used as control to MS360 (Nylander and Roman, 2013). The MS15 and MS360 rats were exposed to the same handling procedures during MS, the only difference being the length of the separation. The separations were performed during the light period and started at 9 AM. The MS procedure has been described in detail elsewhere (Gustafsson and Nylander, 2006). The litters were weighed on PND 0, 3, 7, 10, 13, 16 and the cages were changed on PND 7 and 16. The separations were always performed in the same room and only one person performed all separation and care-taking. The rats subjected to AFR conditions were left undisturbed with the exception of cage change (PND 7, 16) and weighing of the litter (PND 0, 7, 16). These rats were used to assess the possible confounding effect of single housing in the voluntary drinking paradigm. On PND 22 all animals were weaned and then group housed three per cage during adolescence.

### Voluntary Ethanol Drinking

On postnatal week 10 the MS rats were single housed and randomly assigned to water-drinking (MS15W, *n* = 10; MS360W, *n* = 10) or ethanol-drinking groups (MS15E, *n* = 10; MS360E, *n* = 20). More rats were included in the MS360 group based on previous findings of subgroups with responding and non-responding rats regarding ethanol intake (Nylander and Roman, 2013). The AFR rats were assigned to three experimental groups; two groups of rats were single housed and assigned to water (AFRW, *n* = 9) and ethanol drinking (AFRE, *n* = 11), respectively, whereas one group (*n* = 7) was group housed during week 10–16.

The rats exposed to ethanol had free choice between non-sweetened ethanol (5 or 20% made from Ethanol 96%; Solveco AB, Rosersberg, Sweden) and water for three consecutive days a week with ethanol-free days in-between. The first week the rats had free access to 5% ethanol for 24 h and the next week limited access to 5% for 2 h; the following 5 weeks they had access to 20% ethanol in 2 h sessions for 3 days. This drinking paradigm is developed to mimic the common human episodic drinking pattern, with repeated drinking days and non-drinking days in-between (Momeni and Roman, 2014; Palm and Nylander, 2014). Various intermittent models are commonly used in voluntary drinking paradigms to increase the ethanol intake, and intermittent ethanol exposure with ethanol-free days in-between has also been shown to be necessary to induce neurobiological alterations similar to those seen in the transition to habitual and compulsive drinking (Carnicella et al., 2014). Since the current study aimed at investigating early neurobiological markers of risk-drinking correlate, rats were sacrificed at a point where the individual ethanol consumption pattern can be discerned but the voluntary intake associates to the non-addicted state. The limited access restricted to 2 h is an optimal choice to ensure less variation in biological parameters due to individual differences in drinking bouts in a 24 h access paradigm (Carnicella et al., 2014). Ethanol and water were changed every session and the bottle position was altered every day to avoid position preference. At the end of each session, the ethanol and water intake was quantified by weighing the bottles. Care was taken to minimize spillage. After 5 weeks of access to 20% ethanol, the rats weight was measured and thereafter they were decapitated (**Figure 2**). There were no differences in ethanol drinking groups for ethanol intake, as reported in (Comasco et al., 2015). The weekly voluntary ethanol intake in AFR, MS15 and MS360 rats during the last week before decapitation was 1.18 g/kg/2 h (0.62–1.66) in AFR rats, 1.32 g/kg/2 h (0.39–1.77) in MS15 rats, and 1.32 g/kg/2 h (0.60–2.05) in MS360. The ethanol-drinking animals were sacrificed immediately after a 2 h drinking session. Trunk blood was collected from rats randomly chosen from different treatment groups. The hypothalamus and pituitary were removed and immediately frozen on dry ice and stored at -80°C.

### Genetic Analyses

RNA and DNA isolation: RNA and DNA were simultaneously isolated from the rat hypothalamus using AllPrep DNA/RNA/miRNA Universal Kit according to the manufacturer’s protocol (Qiagen AB Sollentuna, Sweden). Quantification of both the nucleic acids was carried out using a Nanodrop ND 1000 spectrometer.

### Gene Expression Analyses

cDNA synthesis: RNA (700 ng) was converted to cDNA using QuantiTect Reverse: Transcription Kit (Qiagen AB Sollentuna, Sweden). The newly synthesized cDNA was diluted 20x and stored at 20°C until further use. gDNA contamination was controlled at three check-points: (1) On column DNase I treatment during the extraction process; (2) gDNA wipe-out reaction prior to cDNA synthesis; and (3) Designing of primers across two adjacent exons to avoid any unspecific amplification of genomic DNA.

Diluted cDNA (20x) was used to assess the expression of *Pomc, Avp, Oxt, Crhr1, Nr3c1, Fkbp5, Mecp2*, and *Dnmt1* in the hypothalamus, and *Pomc, Crhr1, Nr3c1, Fkbp5, Mecp2*, and *Dnmt1* in the pituitary, as well as *Actb, Gapdh* and *Rpl19*, as housekeeping genes, using the CFX96 Touch Real-Time PCR Detection System real time PCR (**Supplementary Table S2**). The *Crh*, *Avp* and *Oxt* gene expression was studied in the hypothalamus where these genes are known to be expressed but not in the pituitary.

Quantification of *Crh* mRNA was performed using TaqMan^®^ Universal Master Mix II, and a TaqMan^®^ Gene Expression Assays for *Crh* (Rn01462137_m1) with a FAM labeled exon spanning probe, and one assay for *Actb* (Rn00667869_m1) and *Gapdh* (Rn01775763_g1) which were used as reference genes. PCR conditions were set following the guidelines provided by the manufacturer (Life technologies Europe BV, Stockholm). Primer specificity was checked using melt curve and gel electrophoresis to verify the amplification of a single product (**Supplementary Figure S1**).

#### Data Analysis

Data of the relative fluorescence unit (RFU) were collected and PCR efficiency and threshold values were calculated using LinregPCR software (http://www.hartfaalcentrum.nl/index.php?main=files&sub=0) (Ruijter et al., 2009). Cq values were adjusted across the plates using correction factors derived from threshold and PCR efficiency values. Samples with normalized Cq values that had a standard deviation of more than 0.5 were excluded. Relative gene transcripts levels were determined using the ΔCT method (Biorad real time PCR application guide). All the laboratory and pre-processing analyses were performed in a blind manner.

### DNA Methylation Analyses

Considering previously shown association of DNA methylation with ELS for *Fkbp5*, *Pomc* and *Avp* (Murgatroyd et al., 2009; Klengel et al., 2013; Wu et al., 2014), as well as the proposed pharmacological relevance of *Crhr1* in AUD treatment (Gehlert et al., 2007), these four genes were chosen to investigate potential DNA methylation differences in their promoter region (**Supplementary Table S2**). The criteria used to select the target amplicons were the presence of Transcription Factor Binding Sites (TFBSs) and/or CpG islands in the promoter region, preferably close to the Transcription Start Site. In addition, previous studies that investigated DNA methylation patterns of the genes of interest were considered. Regarding the *Pomc* gene, the selected amplicon was a 268 bp long region comprising a CpG island and many TFBSs (**Supplementary Figure S2**). The target sequences are reported in **Supplementary Table S2**; **Supplementary Figure S2**.

The bisulphite quantitative pyrosequencing technique was used to assess the methylation pattern of the four genes using 500 ng of DNA at a concentration of 20 ng/μl (EpigenDx (Hopkinton, MA, USA) (Supplementary Material). To estimate errors, 10% of the samples (9 out of 81) were duplicated in each assay. No significant difference was present between duplicates as assessed by the Wilcoxon signed-rank test. The average difference between the duplicates for any gene was <2%, indicating a 98% replicability of each gene assay. To verify the efficiency of sodium bisulfite DNA conversion, each individual pyrosequencing reaction included a non-CpG cytosine as an internal bisulfite modification control, while low, medium, and high methylated DNA samples were included as controls in each plate. Also, 0, 5, 10, 25, 50, 75 and 100% methylated DNA samples obtained by mixing unmethylated and *in vitro* methylated DNA were used as standards during the first strand synthesis PCR and pyrosequencing analysis. The percent methylation obtained from the study showed high correlation when compared with expected methylation percentages where correlation coefficient of 0.96 for *Avp*, 0.97 for *Crhr1*, 0.98 for *Fkbp5*, and 0.94 for *Pomc*, indicating high quality of the data.

#### Data Analysis

Since the unmethylated CpG sites were converted into uracil, the percentage of methylation was computed by calculating the ratio between both C and T peaks in the pyrogram at the same position using the QCpG software. The calculated percentage was ranging between 100%, indicating the highest methylation, and 0%, indicating no methylation.

### Hormone Analysis

Samples were analyzed using the commercial ImmuChem^TM^ Double Antibody Corticosterone ^125^I RIA kit for rats and mice (MP Biomedicals LLC, Orangeburg, NY, USA) in accordance with the included protocol, with the exception of the addition of one standard (12.5 ng/ml). All samples were analyzed in duplicates. According to the protocol of the RIA kit, the intra-assay variation was 4.4–10.3% and the inter-assay variation 6.5–7.2%. The corticosterone antiserum showed 100% cross-reactivity with corticosterone, while cross-reactivity to other steroids was 0.34% to deoxycorticosterone, 0.10% to testosterone, 0.05% to cortisol, and <0.05% to other tested steroids. Corticosterone levels of the MS rats have been previously reported (Bendre et al., 2015).

### Statistical Analyses

To study the effect of ethanol and housing on gene expression, DNA methylation, and corticosterone, between-groups analyses were performed using the Mann–Whitney *U* test. Mann–Whitney *U* test was chosen because most of the data for gene expression, DNA methylation (except average methylation) and corticosterone were not normally distributed. Additionally, the GLM two-way ANOVA test with type III sum of squares, which is considered robust to violations of normality (Glass et al., 1972), was performed to assess the interaction and main effects of ELS and ethanol. The Mann–Whitney *U* test was performed to test group differences between MS groups according to the research questions, i.e., water drinking MS360 vs. water drinking MS15 (effect of ELS), ethanol vs. water drinking MS15 (ethanol-induced effect in MS15 rats), and ethanol vs. water drinking MS360 rats (ethanol-induced effect in MS360 rats). To provide results comparable to the previous literature using AFR animals as controls in MS paradigms, main and interaction effects of MS and ethanol were also assessed in MS360 compared to AFR rats.

Bivariate correlation tests were performed using the Spearman’s rank test. Bonferroni correction for multiple testing was applied. There were performed 105 gene expression vs. gene expression correlations tests; 15 gene expression vs. ethanol intake, and 15 gene expression vs. corticosterone tests, *Avp*: 5, *Crhr1*: 14, *Fkbp5*: 7, and *Pomc*: 17 gene methylation vs. expression, 50 gene methylation vs. ethanol intake and 50 gene methylation vs. corticosterone correlation tests.

## Results

### Effects of Single Housing and Voluntary Ethanol Intake in AFR Rats

The effect of single housing and ethanol drinking on gene expression, DNA methylation and corticosterone levels was examined in conventionally reared AFR rats in order to understand how different housing paradigms affect the HPA axis.

#### Gene Expression (**Figure 3**)

**FIGURE 3:**
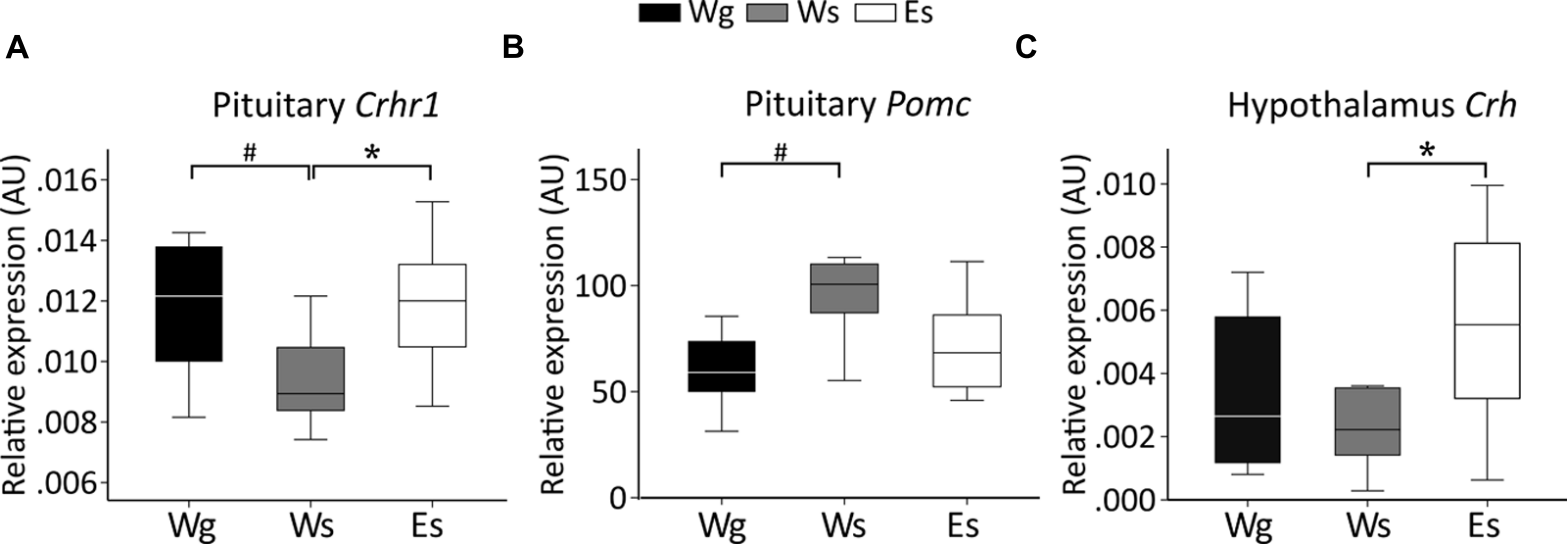
**Relative gene expression (median) in animal facility reared rats.** Between-group comparisons indicate an effect of voluntary ethanol drinking on **(C)** *Crh* in the hypothalamus (*U* = 20; *p* = 0.043) and **(A)** *Crhr1* (*U* = 17; *p* = 0.012) in the pituitary and an effect of single housing on **(A)** *Crhr1* (*U* = 14; *p* = 0.036) and **(B)** *Pomc* (*U* = 14; *p* = 0.036) in the pituitary. AU, arbitrary units; *Crh*, corticotropin releasing hormone; *Crhr1*, corticotropin releasing hormone receptor 1; E, ethanol drinking; g, group housed; *Pomc*, proopiomelanocortin; s, single housed; W, water drinking.^#^*p* ≤ 0.05: effect of single housing; ^∗^*p* ≤ 0.05: effect of ethanol.

Single-housed AFR rats had lower *Crhr1* expression (**Figure 3A**) but higher *Pomc* expression (**Figure 3B**) in the pituitary gland, compared to group-housed AFR rats. Voluntary ethanol intake was associated with higher *Crh* expression in the hypothalamus (**Figure 3C**), and *Crhr1* expression in the pituitary (**Figure 3A**), in comparison with water drinking AFR rats.

#### Promoter DNA Methylation (**Figure 4**)

**FIGURE 4:**
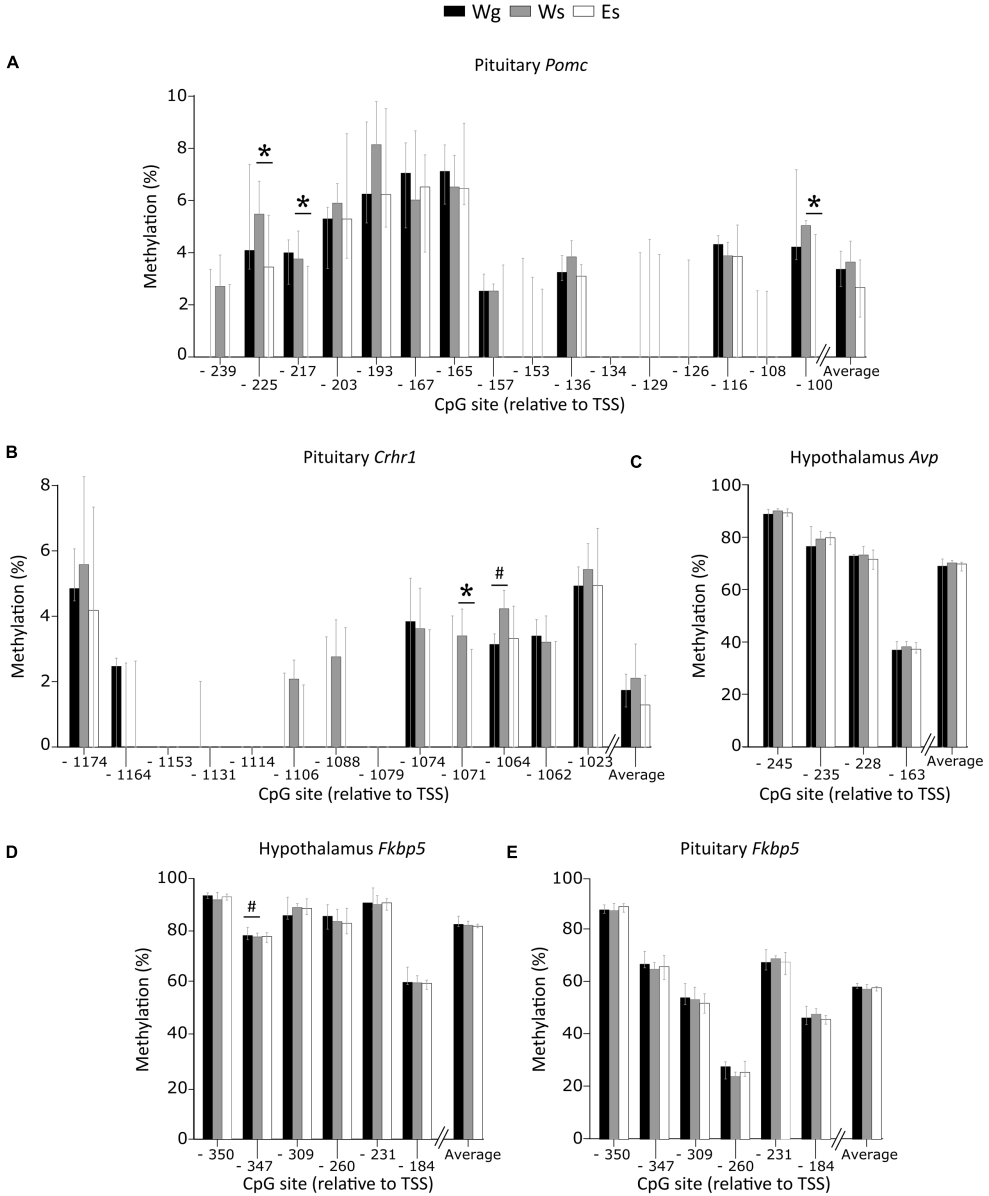
**Percent DNA methylation on single CpG sites and average in the AFR rats.** Between-group comparisons indicate an effect of voluntary ethanol drinking on methylation of **(A)** *Pomc* CpG -225 (*U* = 15; *p* = 0.007), -217 (*U* = 20; *p* = 0.025), -100 (*U* = 20.5; *p* = 0.025), and **(B)** *Crhr1* CpG -1071 (*U* = 22; *p* = 0.038) in the pituitary. An effect of single housing was observed on methylation of **(B)** *Crhr1* CpG -1064 in the pituitary (*U* = 15.5; *p* = 0.024), and **(D)** *Fkbp5* CpG -347 in the hypothalamus (*U* = 7; *p* = 0.029). No effect of ethanol or single housing was observed on the methylation of **(C)** *Avp* in the hypothalamus or **(E)** *Fkbp5* in the pituitary. Bars represent median % methylation and 95% confidence interval. *Avp*, arginine vasopressin; CpG, C—phosphate—G; *Crhr1*, corticotropin releasing hormone receptor 1; E, ethanol drinking; *Fkbp5*, FK506 binding protein 5; g, group housed; *Pomc*, proopiomelanocortin; s, single housed; TSS, transcription start site; W, water drinking ^#^*p* ≤ 0.05: effect of single housing; ^∗^*p* ≤ 0.05: effect of ethanol.

Single housing alone affected methylation patterns with higher methylation of *Crhr1* CpG -1064 in the pituitary (**Figure 4B**), and lower methylation of *Fkbp5* CpG -347 in the hypothalamus (**Figure 4D**), compared to group housed AFR rats. In single housed animals, ethanol drinking AFR rats had lower methylation of *Pomc* CpG -225, -217, and -100 (**Figure 4A**) and *Crhr1* CpG -1071 (**Figure 4B**) in the pituitary compared to water drinking AFR rats. Neither single housing nor ethanol intake showed an effect on average DNA methylation of any gene. Promoter DNA methylation of *Avp* in the hypothalamus (**Figure 4C**) and *Fkbp5* in the pituitary (**Figure 4E**) were not associated with either ethanol drinking or single housing.

#### Corticosterone (**Figure 5**)

**FIGURE 5:**
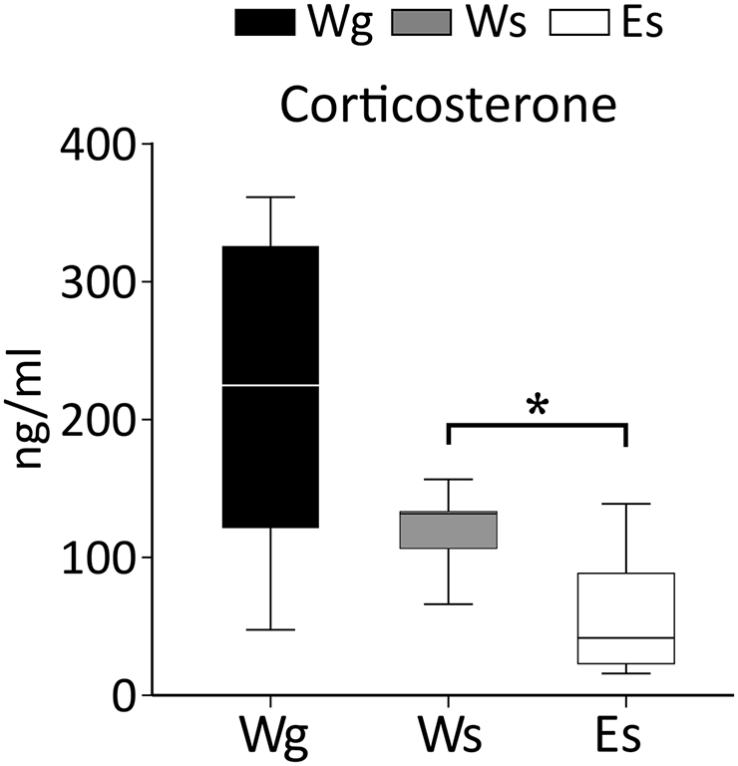
**Corticosterone levels in AFR rats.** The results indicate an effect of ethanol on corticosterone (*U* = 14; *p* = 0.010), E, ethanol drinking; g, group housed; s, single housed; W, water drinking. ^∗^: Mann–Whitney test *p* ≤ 0.05.

Single housing in adulthood was not associated with differences in corticosterone levels in AFR rats. Ethanol compared to water drinking AFR rats displayed lower corticosterone. The group housed rats displayed larger within-group variability than the other groups. Such variability was not attributable to diurnal variation because there was no effect of time of day when the rats were decapitated. However, differences in corticosterone levels were observed between the first rat taken from the cage compared to the second and the last rat remaining in the cage.

### Effects of ELS and Voluntary Ethanol Intake in MS Rats

To understand how ELS affects genes involved in the HPA axis as well as the interaction with ethanol the main effects of ELS, ethanol drinking, and their interaction, on gene expression, DNA methylation, and corticosterone levels were examined in adult MS rats.

#### Gene Expression (**Figure 6**)

**FIGURE 6:**
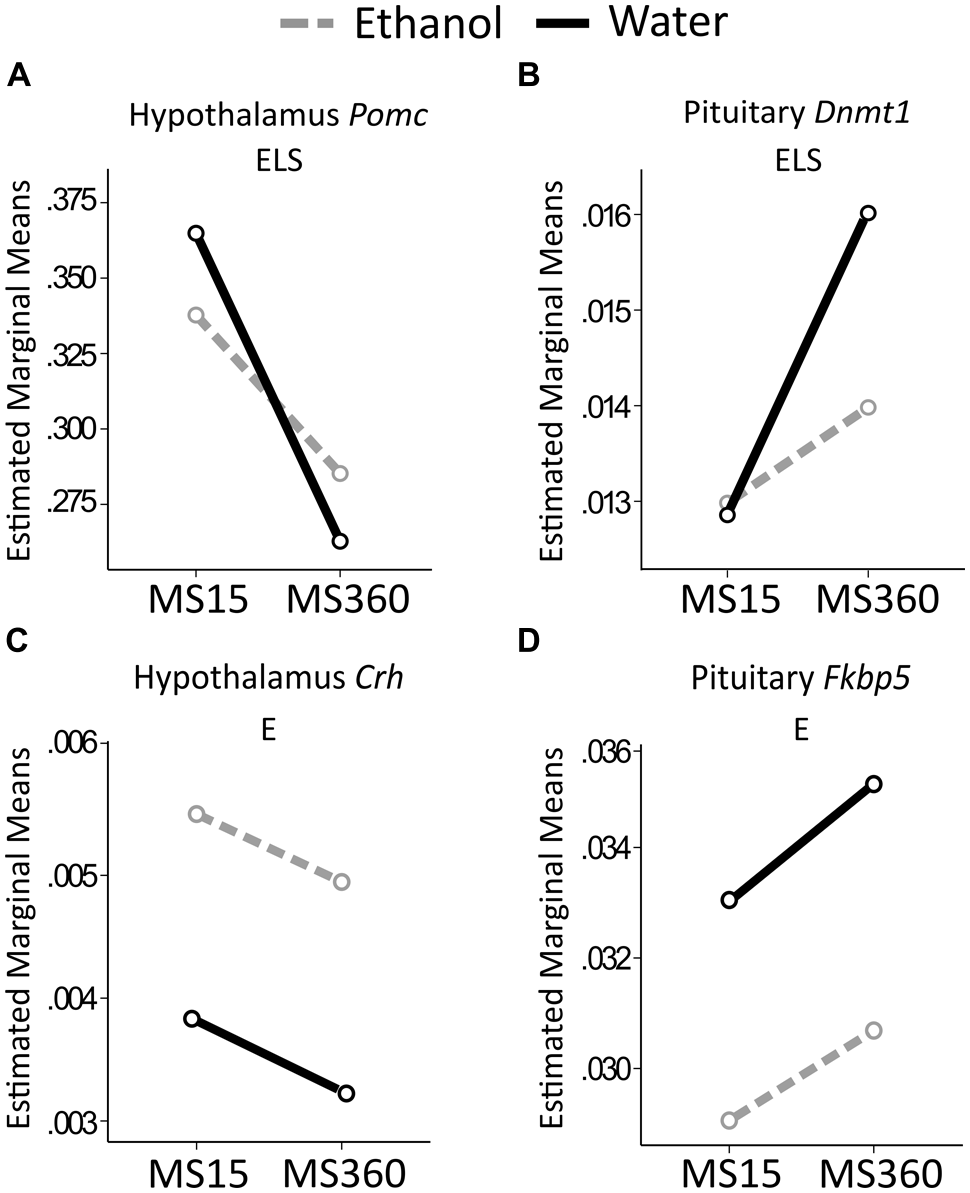
**Relative gene expression in the MS rats.** The two way ANOVA type III sum of squares test revealed a main effect of ELS on **(A)** *Pomc* in the hypothalamus (*F* = 5.806; *p* = 0.020; Adj. R^2^ = 0.060) and **(B)** *Dnmt1* in the pituitary (*F* = 10.316; *p* = 0.002; Adj. R^2^ = 0.175). A main effect of ethanol (E) was found on **(C)** Crh in the hypothalamus (*F* = 4.092; *p* = 0.049; Adj. R^2^ = 0.026), and **(D)** *Fkbp5* in the pituitary (*F* = 4.495; *p* = 0.039; Adj. *R*^2^ = 0.039). *Crh*, Corticotropin releasing hormone; *Dnmt1*, DNA methyltransferase (cytosine-5) 1; E, ethanol; ELS, early life stress; *Fkbp5*, FK506 binding protein 5; MS, maternal separation for 15/360 min; *Pomc*, proopiomelanocortin.

Early life stress had a main effect on *Pomc* expression in the hypothalamus where MS360 rats had lower expression than MS15 rats, irrespectively of drinking water or ethanol (**Figure 6A**). Furthermore, a main effect of ELS was present on expression of *Dnmt1* in the pituitary (**Figure 6B**) where MS360 rats had higher *Dnmt1* expression than MS15 rats. A focused between-group analysis revealed that water drinking MS360 rats had higher *Dnmt1* expression in the pituitary compared to water drinking MS15 rats (*U* = 21, *p* = 0.029). Furthermore, ethanol drinking MS360 rats had lower *Dnmt1* expression (*U* = 44, *p* = 0.013) compared to water drinking MS360 rats whereas ethanol drinking had no impact on expression in the MS15 rats (**Figure 6B**). A main effect of ethanol intake was associated with higher *Crh* expression in the hypothalamus (**Figure 6C**) and lower *Fkbp5* expression in the pituitary (**Figure 6D**) in ethanol drinking rats irrespective of early life rearing condition. No interaction effect was observed on expression of any gene studied.

#### Promoter DNA Methylation (**Figure 7**)

**FIGURE 7:**
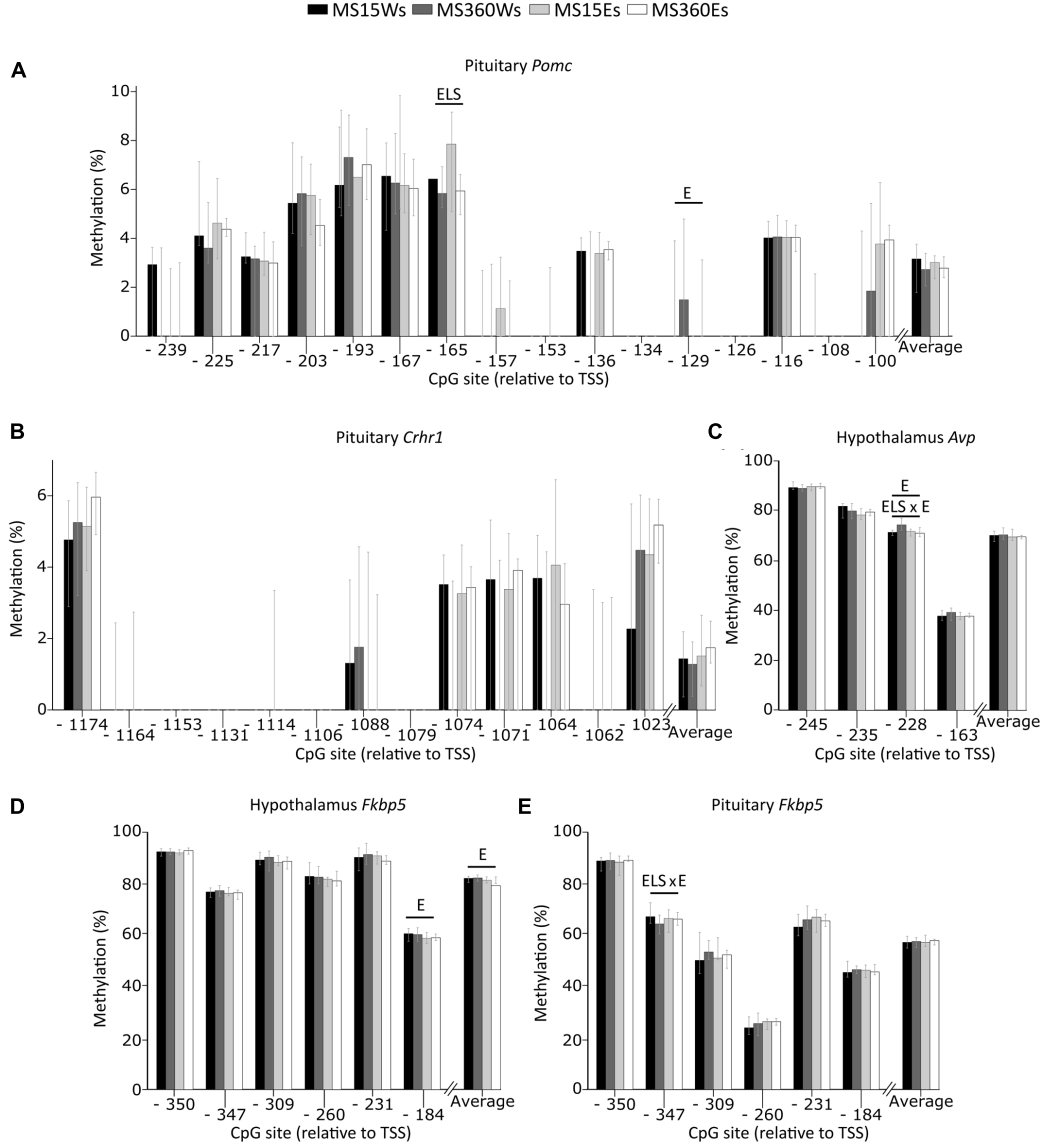
**Percent DNA methylation at single CpG sites and average in the MS rats.** The two way ANOVA type III sum of squares test revealed a main effect of ELS on methylation of **(A)** *Pomc* CpG -165 in the pituitary (*F* = 7.473; *p* = 0.009; Adj. *R*^2^ = 0.097) as well as a main effect of ethanol (E) on methylation of **(A)** *Pomc* CpG -129 (*F* = 4.766; *p* = 0.034; Adj. *R*^2^ = 0.082) in the pituitary and **(C)** *Avp* CpG -228 (*F* = 4.081; *p* = 0.049; Adj. *R*^2^ = 0.216), **(D)** *Fkbp5* CpG -184 (*F* = 5.104; *p* = 0.029 Adj. *R*^2^ = 0.043) as well as average **(D)** *Fkbp5* methylation (*F* = 5.547; *p* = 0.023; Adj. *R*^2^ = 0.060) in the hypothalamus. An interaction between ELS and ethanol effect (ELS × E) was observed on methylation of **(C)** *Avp* CpG -228 in the hypothalamus (*F* = 9.578; *p* = 0.003; Adj. *R*^2^ = 0.216), and **(E)** *Fkbp5* CpG -347 in the pituitary (*F* = 5.777; *p* = 0.020; Adj. *R*^2^ = 0.082). No effect of stress or ethanol was observed on the methylation of **(B)** *Crhr1* in the pituitary. Bars represent median % methylation and 95% confidence interval *Avp*, arginine vasopressin; CpG, C—phosphate—G; *Crh*, corticotropin releasing hormone; *Crhr1*, corticotropin releasing hormone receptor 1; E, ethanol; *Fkbp5*, FK506 binding protein 5; *Pomc*, proopiomelanocortin; ELS, early life stress; TSS, transcription start site.

In the pituitary, a main effect of ELS was associated with lower *Pomc* CpG -165 DNA methylation in MS360 than MS15 rats (**Figure 7A**). This effect was mainly driven by the lower methylation on *Pomc* CpG -165 in ethanol drinking MS360 compared to MS15 rats (*U* = 51, *p* = 0.031). Water drinking MS360 rats had higher methylation of *Avp* -228 compared to MS15 (*U* = 6, *p* < 0.001). Furthermore, ethanol intake had a main effect on *Pomc* CpG -129 where ethanol drinking rats had lower methylation compared to water drinking rats (**Figure 7A**). A main effect of ethanol intake was present in the hypothalamus on *Avp* CpG -228, and there was also an interaction between ELS and ethanol intake for *Avp* CpG -228 methylation driven by lower methylation in ethanol drinking MS360 compared to water drinking MS360 rats (*U* = 27, *p* = 0.001) (**Figure 7C**). *Fkbp5* CpG -184 (**Figure 7D**), and average *Fkbp5* methylation in the hypothalamus was associated with ethanol drinking rats having lower methylation compared to water drinking rats (**Figure 7D**). Furthermore an interaction effect between ELS and ethanol intake was observed on *Fkbp5* CpG -347 in the pituitary (**Figure 7E**) indicating that ethanol drinking results in opposite effects in MS15 and MS360 rats. No difference in the promoter DNA methylation of *Crhr1* in the pituitary was observed (**Figure 7B**).

#### Corticosterone (**Figure 8**)

**FIGURE 8:**
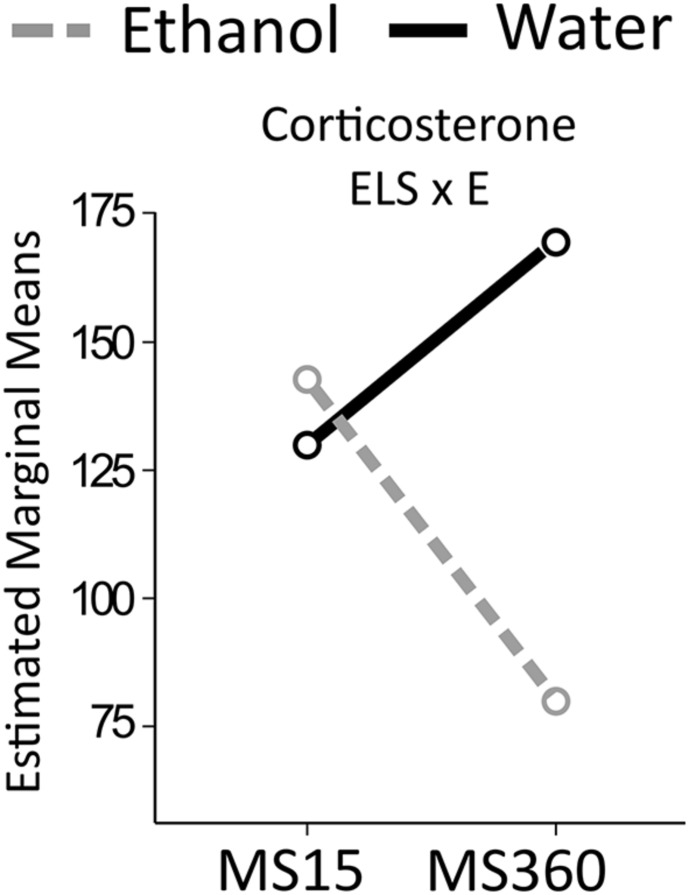
**Serum corticosterone levels in the MS rats.** The two way ANOVA type III sum of squares test revealed an interaction effect between ELS and ethanol (ELS × E) on corticosterone (*F* = 5.314; *p* = 0.027; Adj. *R*^2^ = 0.178). MS, maternal separation for15/360 min.

An interaction effect between ELS and ethanol was found on corticosterone levels. Ethanol drinking MS360 rats had lower corticosterone levels (*U* = 20, *p* = 0.003) compared to water drinking MS360 rats, whereas no difference was noted between water and ethanol drinking MS15 rats. Ethanol drinking MS360 rats also had lower levels than ethanol drinking MS15 rats (*U* = 22, *p* = 0.033).

Body weight of the water drinking rats exposed to ELS was lower compared to MS15 rats (*U* = 6, *p* < 0.001). However, body weight as a covariate did not influence the results.

### Correlations

Correlation analyses were performed separately in each group to assess the effect of ELS or voluntary drinking on (i) correlations amongst expression of the genes, and (ii) correlations between gene expression, promoter methylation, ethanol intake and corticosterone.

#### Expression vs. Expression (**Table 1**)

**Table 1:** Group-wise bivariate correlation between expression of genes.

	Gene 1	Gene 2	Region	Group	*r_s_*	*p*
HPA axis - HPA axis	*Crhr1*	*Nr3c1*	Hypothalamus	MS360 Es	0.878	3.57E-07
	*Crhr1*	*Nr3c1*	Hypothalamus	MS360 Ws	0.903	3.44E-04
	*Crhr1*	*Oxt*	Hypothalamus	MS15 Ws	0.903	3.44E-04
	*Crhr1*	*Oxt*	Hypothalamus	MS360 Ws	0.964	7.32E-06
	*Oxt*	*Avp*	Hypothalamus	MS360 Es	0.753	2.01E-04
HPA axis - DNA methylation	*Crhr1*	*Dnmt1*	Hypothalamus	MS15 Ws	0.952	2.28E-05
	*Crhr1*	*Dnmt1*	Hypothalamus	MS360 Es	0.798	2.44E-05
	*Crhr1*	*Dnmt1*	Hypothalamus	MS360 Ws	0.952	2.28E-05
	*Nr3c1*	*Dnmt1*	Hypothalamus	AFR Es	0.927	1.12E-04
	*Nr3c1*	*Dnmt1*	Hypothalamus	MS15 Ws	0.952	2.28E-05
	*Nr3c1*	*Dnmt1*	Hypothalamus	MS360 Es	0.949	1.88E-10
	*Nr3c1*	*Dnmt1*	Hypothalamus	MS360 Ws	0.964	7.32E-06
	*Crhr1*	*Mecp2*	Hypothalamus	AFR Es	0.927	3.97E-05
	*Crhr1*	*Mecp2*	Hypothalamus	MS360 Ws	0.939	5.48E-05
	*Oxt*	*Mecp2*	Hypothalamus	MS360 Ws	0.939	5.48E-05
	*Nr3c1*	*Mecp2*	Pituitary	MS360 Es	0.805	1.90E-05


*AFR, animal facility reared*;* E, ethanol drinking; g, group housed; MS, maternal separation (15 or 360 min); *r_s_*, spearman’s correlation coefficient; s, single housed; W, water drinking.*

After Bonferroni correction, strong positive correlations, mainly restricted to the hypothalamus and ELS group, were observed amongst expression of HPA receptor (*Nr3c1, Crhr1*) and methylation-related genes (*Dnmt1* and *Mecp2*).

#### Expression vs. Methylation, Ethanol Intake and Corticosterone

After Bonferroni correction, gene expression was not correlated with average gene methylation, except in water drinking MS15 rats where *Pomc* expression in the pituitary was positively correlated with its average methylation (*r*_s_ = 0.903; *p* < 0.001). Regarding methylation at single CpG sites, in group housed AFR rats methylation of *Fkbp5* CpG -335 in the hypothalamus (*r*_s_ = 0.943, *p* = 0.005) and *Fkbp5* CpG -184 in the pituitary (*r*_s_ = 0.881, *p* = 0.004) was positively correlated with *Fkbp5* expression in the respective brain regions. In ethanol drinking MS360 rats, methylation of *Fkbp5* CpG -376 positively correlated with its expression in the pituitary (*r*_s_ = 0.636, *p* = 0.003). Gene expression was not correlated with ethanol intake at postnatal week 15 for any gene studied, after Bonferroni correction. A strong positive correlation surviving correction for multiple testing was noted between *Dnmt1* expression in the hypothalamus and corticosterone levels only in ethanol drinking MS15 rats (*r*_s_ = 1.000; *p* < 0.001).

#### Methylation vs. Ethanol Intake and Corticosterone

There were no significant correlations, after Bonferroni correction, in any of experimental group between average DNA methylation of any gene and ethanol intake at postnatal week 15, likewise with corticosterone levels.

### Comparison of Main and Interaction Effects Using Different Reference Groups

**FIGURE 9:**
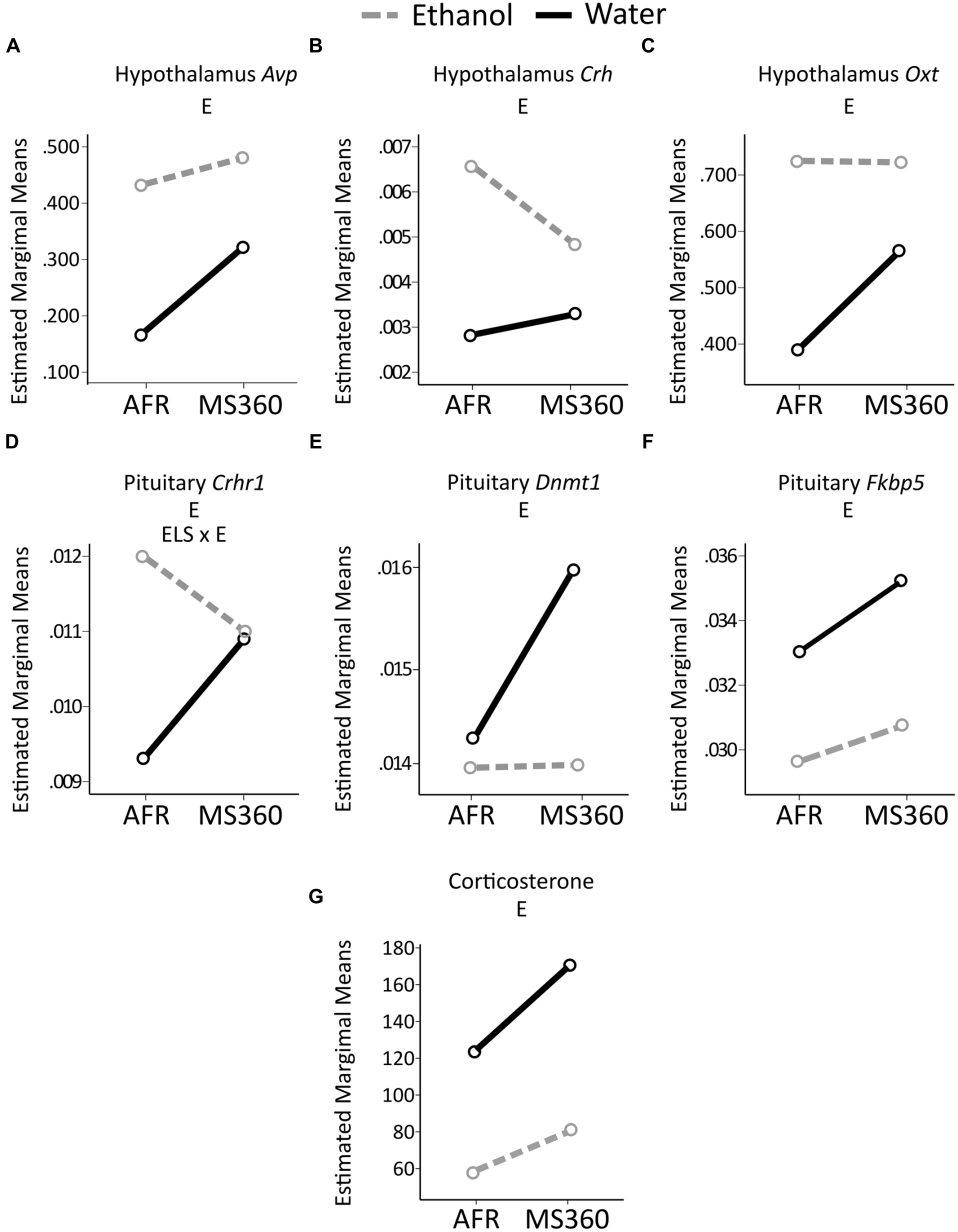
**Relative gene expression in the MS360 and AFR rats.** The two way ANOVA type III sum of squares test indicates a main effect of ethanol (E) on expression of **(A)** Avp (*F* = 8.44, *p* = 0.006 Adj. *R*^2^ = 0.138), **(B)** *Crh* (*F* = 9.17, *p* = 0.004, Adj. *R*^2^ = 0.129) and **(C)** *Oxt* (*F* = 4.39, *p* = 0.042 Adj. *R*^2^ = 0.043) in the hypothalamus as well as on expression on **(D)** *Crhr1* (*F* = 6.00, *p* = 0.018, Adj. *R*^2^ = 0.114), **(E)** *Dnmt1* (*F* = 4.79, *p* = 0.034, Adj. *R*^2^ = 0.115) and **(F)** *Fkbp5* (*F* = 4.71, *p* = 0.035, Adj. *R*^2^ = 0.046) in the pituitary **(G)** corticosterone levels (*F* = 22.26, *p* < 0.001, Adj. *R*^2^ = 0.330) and an interaction effect between ELS and ethanol (ELS × E) on expression of **(D)** *Crhr1* (*F* = 4.58, *p* = 0.038, Adj. *R*^2^ = 0.114). AFR, animal facility reared; MS, maternal separation for 360 min.

In the literature, MS paradigms with either AFR or MS15 as controls to prolonged MS can be found. Therefore, ELS and ethanol drinking effects were also tested in MS360 compared to AFR rats. Irrespectively of reference group (i.e., MS15 or AFR) a main effect of ethanol intake was found on expression of *Fkbp5* in the pituitary and *Crh* in the hypothalamus as well as no main effect of ELS on corticosterone levels (**Table 2**). The rest of the results differed depending on the control group used, with more significant associations being present when testing the comparison MS360 vs. AFR rats (**Table 2**, **Figures 6** and **9**).

**Table 2:** Comparison between group differences using two different reference groups, MS15 or AFR conditions.

Effect	Measurement	Gene (Region)	MS15 and MS360 Direction	AFR and MS360 Direction
ELS	Expression	*Dnmt1* (pit)	↑ in MS360	*–*
		*Pomc* (hyp)	↓ in MS360	*–*
	Hormone	Corticosterone	*–*	*–*
Ethanol	Expression	***Fkbp5*** (pit)	↓**in ethanol drinking**	↓**in ethanol drinking**
		***Crh*** (hyp)	↑ **in ethanol drinking**	↑ **in ethanol drinking**
		*Avp* (hyp)	*–*	↑ in ethanol drinking
		*Dnmt1* (pit)	*–*	↓ in ethanol drinking
		*Oxt* (hyp)	*–*	↑ in ethanol drinking
		*Crhr1* (pit)	*–*	↑ in ethanol drinking (in AFR)
	Hormone	Corticosterone	–	↓ in ethanol drinking
ELS × ethanol	Expression	*Crhr1* (pit)	*–*	↑ in AFR, ethanol drinking (no difference in MS360)
	Hormone		↓ in MS360, ethanol drinking (no difference in MS15)	*–*


*The bold represents consistent results across the two comparisons. AFR, animal facility reared; E, ethanol drinking; ELS, early life stress; g, group housed; hyp, hypothalamus; MS, maternal separation; pit, pituitary; W, water drinking.*

## Discussion

### Effects of Single Housing and Voluntary Ethanol Intake in AFR Rats

The effect of housing was assessed by comparing single and group housed water drinking rats, whereas the effect of voluntary ethanol drinking was assessed by comparing single housed ethanol *vs.* water drinking AFR rats. Single housing was associated with down-regulation of *Crhr1* and up-regulation of *Pomc* in the pituitary whereas ethanol drinking was associated with up-regulation of *Crhr1*. Single housing stress during adolescence associates with increased basal ACTH levels, which is derived from *Pomc* (Weiss et al., 2004), and increased ethanol intake (Wolffgramm, 1990). Furthermore, housing dependent effects of alcohol have been observed on opioid peptides in both adolescent and adult rats (Palm and Nylander, 2014).

On the other hand, single housing has been previously associated with lower ethanol intake in Sprague Dawley rats (Doremus et al., 2005) or no effect on ethanol intake in adult Long Evans or Wistar rats (Schenk et al., 1990; Thorsell et al., 2005). Moreover, single housing has been shown to influence molecular profiles despite having no effect on the ethanol intake (Thorsell et al., 2005). The up-regulation of *Crhr1* expression in the pituitary in ethanol drinking rats is in line with the proposed use of *Crhr1* antagonism as a treatment for AUD (Funk et al., 2007; Gehlert et al., 2007; Spanagel et al., 2014). However, a recent clinical trial observed no effect of a *Crhr1* antagonist on neuroendocrine correlates or behavior in AUD patients (Kwako et al., 2015).

The higher *Crh* expression observed in the hypothalamus in ethanol drinking rats is likely a result of adaptive mechanisms, suggested by the observed lower corticosterone levels. The finding is in agreement with previous reports showing increased *Crh* expression in the hypothalamus following acute or short-term ethanol exposure in adult rats (Madeira and Paula-Barbosa, 1999).

Neither single housing nor ethanol drinking was associated with *Avp* or *Oxt* expression in the hypothalamus. Chronic ethanol vapor exposure has been associated with both lower (Sanna et al., 1993) and higher *Avp* expression (Logrip et al., 2013), whereas prolonged oral ethanol did not affect mRNA expression in the paraventricular nucleus (Silva et al., 2002). Lower *Oxt* peptide levels have been detected in the hypothalamus of mice exposed to single compared to group housing in adulthood (Liu et al., 2013).

Regarding *Fkbp5*, *Nr3c1*, *Mecp2*, and *Dnmt1*, no effect of ethanol or housing was found. Finally, both ethanol and single housing affected methylation at single CpG sites; however, whether these small differences (<5%) have a biological impact remains unknown.

### Effects of ELS and Voluntary Ethanol Intake in MS Rats

Prolonged MS induces long-term changes in various neurotransmitter systems (Gudsnuk and Champagne, 2012; Harrison and Baune, 2014; Maccari et al., 2014), and relates to propensity for excessive voluntary ethanol intake (Roman and Nylander, 2005). Although previous studies support long-term alterations of the HPA axis after MS, the exact nature of the impact of ELS is not understood (Lehmann and Feldon, 2000; Pryce and Feldon, 2003).

#### Effect of ELS in MS Rats

Down-regulation of *Pomc* in the hypothalamus was associated with ELS, but no effect was observed on *Crh* or *Crhr1* expression. MS has been associated with increased expression of *Crh* in the hypothalamus of rats (Plotsky and Meaney, 1993; Huot et al., 2004; Plotsky et al., 2005) and *Pomc* in the pituitary of mice (Wu et al., 2014). The contradictory results for *Crh* and *Pomc* might be due to difference in the duration of MS (180 vs. 360 min), group vs. single housing in adulthood, and species (mice vs. rats) or strains (Long-Evans vs. Wistar). As the product of *Pomc* mRNA is a prohormone which is cleaved into tissue specific peptides (e.g., ACTH, beta-endorphin) with diverse physiological functions (Smith and Funder, 1988), changes in *Pomc* gene expression can relate to other physiological conditions than stress. In the present study, ELS had no effect on *Avp* expression and on methylation of the *Avp* promoter region, in line with findings on mRNA (Veenema et al., 2006) and peptide levels (Oreland et al., 2010). However, Murgatroyd et al. (2009) reported that mice subjected to MS180 had higher mRNA expression of *Avp* and *Nr3c1* as well as hypomethylation of an enhancer region of *Avp* in the hypothalamus compared to AFR animals. Combination of methodological differences discussed earlier for *Crh* and *Pomc* as well as different genomic loci assessed for methylation herein might explain discordant results. Regarding *Fkbp5* or *Oxt* no effect of ELS was observed.

#### Effects of Voluntary Ethanol Drinking in MS Rats

All voluntary ethanol drinking rats had higher *Crh* expression in the hypothalamus. Acute ethanol exposure has been reported to increase *Crh* expression in the hypothalamus of conventionally reared rats (Madeira and Paula-Barbosa, 1999). This finding is consistent with what observed in AFR rats, indicating a robust effect of ethanol on *Crh* expression irrespective of rearing condition.

Ethanol drinking rats also had lower *Fkbp5* expression in the pituitary irrespective of MS condition. However, the absence of an effect of ethanol drinking in AFR rats suggests *Fkbp5* mediated mechanism following exposure to ethanol in the pituitary to be restricted to handling of pups performed in the MS procedure. Moreover, a main effect of ethanol was found on average *Fkbp5* promoter methylation in the hypothalamus, but *Fkbp5* promoter methylation was not correlated with its gene expression. Reports of ethanol-induced effects on *Fkbp5* in HPA axis are lacking. Corticosterone levels in MS360, but not in MS15, rats were sensitive to ethanol as evidenced by lower levels detected only in MS360 rats.

No main effect of ethanol was found on *Avp*, *Crhr1*, *Oxt*, *Nr3c1*, and *Pomc* or DNA methylation genes, *Dnmt1* and *Mecp2*. However, between-group analyses revealed that rats exposed to ELS displayed higher *Dnmt1* expression in the pituitary compared to controls and that ethanol drinking in these rats resulted in lower *Dnmt1* expression, i.e., more close to MS15 rats. This indicates an opposite effect of ethanol drinking and ELS on the methylation machinery. Moreover, in ethanol drinking MS15 rats, *Dnmt1* expression in the hypothalamus was positively correlated with corticosterone levels. Corticosterone treatment of pituitary cells has been reported to decrease *Dnmt1* expression in a dose dependent manner (Yang et al., 2012) thus a negative correlation can be expected. MS15 is suggested to be protective against risk of AUD (Nylander and Roman, 2013; Nishi et al., 2014). Whether the positive correlation observed herein is underlying a protective mechanism against AUD in MS15 rats needs further investigation. Also, the positive correlation between average methylation and *Pomc* expression only in water drinking MS15 rats might also indicate a possible underlying mechanism against behavioral abnormalities in adulthood.

#### DNA Methylation and HPA Axis

In the current study ELS was associated with higher *Dnmt1* expression in the pituitary. *In vitro* mice hippocampal cells challenged with corticosterone showed a dose-dependent decrease in *Dnmt1* expression indicating a possible regulatory role in stress physiology (Yang et al., 2012). No effect of ELS was observed on *Mecp2*. Finally, most of the correlations between genes were found in the hypothalamus and in the MS360 group. The correlations between the expression of HPA receptors (*Nr3c1*, *Crhr1*) and DNA methylation regulatory genes (*Dnmt1*, *Mecp2*) in the hypothalamus of M360 rats indicate possible epigenetic regulation of the two receptor genes upon exposure to ELS. These correlations thus call for further studies on the molecular mechanisms underlying ELS mediated vulnerability to ethanol drinking.

### Methodological Considerations

A well validated MS model was employed to investigate long-term consequences of ELS (Nylander and Roman, 2013). Herein, MS15 was the control based on the notion that short periods of MS are associated with adult low ethanol consumption (Moffett et al., 2007; Nylander and Roman, 2013) as well as it controls for the handling effect. However, the outcome of prolonged separations are depending on the control group (Lehmann and Feldon, 2000; Pryce and Feldon, 2003; Nylander and Roman, 2013), and therefore AFR was also included to improve the comparability with previous studies. When AFR was used as control, more effects of ethanol and ELS were found on gene expression, and only two findings were consistent irrespectively of the control group. The higher number of significant differences in gene expression when AFR animals were used as controls was expected due to the different handling conditions in the two controls (AFR and MS15) and the constant presence of the dam (AFR) vs. the more naturalistic short periods of maternal absence (MS15). The comparison was performed to test a methodological consideration regarding the choice of the control group. These results thus highlight the importance of choosing the control group, and confirm previous reported effects of handling *per se* (Pryce and Feldon, 2003; Jaworski et al., 2005; Nylander and Roman, 2013). The lower body weight after MS accompanied by higher corticosterone levels observed in the present study has been previously demonstrated (Kalinichev et al., 2002). However, body weight increase was similar between groups over time and it had no effect on the gene expression or corticosterone levels when used as a covariate in the general linear model. Corticosterone levels depend on circadian rhythm and single measurement is a limitation of the present study. On the other hand, the sampling of blood for the measurement of corticosterone levels at the same time of the sampling of the brain for gene expression and DNA methylation analyses makes the correlation tests of relevance.

Moreover, the investigation of the effect of housing allowed detecting possible effect of single housing, a factor often overlooked. Another strength of the present study is the gene set based approach which provides a comprehensive view of effects of ELS and ethanol consumption on the HPA axis. To gain a deeper understanding, proteomic analyses as well as analysis of hypothalamic subnuclei would have been an optimal complement.

Regarding the ethanol intake, the majority of the rats consumed 1–2 g/kg, during the 2 h session. Even though this level of consumption is moderate compared to that of genetically alcohol-preferring rats or rats in the addictive state, it relates to a binge intake that is defined as “a pattern of drinking that brings blood alcohol concentration levels to 0.08 g/dL in about 2 h” according to NIAAA and that clearly has neurobiological effects. However, the purpose of the present study was to investigate early neurobiological signatures related to voluntary alcohol intake in habitual drinking non-preferring rats. In fact, molecular changes concomitant to initial ethanol drinking are likely to contribute to the development of AUD (Koob, 2013). The voluntary and episodic drinking paradigm used herein simulates human episodic drinking patterns (Crabbe et al., 2011; Carnicella et al., 2014). The use of outbred rats gives further strength for translational interpretations since they represent a heterogeneous population compared to alcohol-preferring strains of rodents.

## Conclusion

This study provides a comprehensive investigation of stress and DNA methylation regulatory genes. Ethanol displayed opposite effects compared to single housing indicating ethanol drinking to alleviate stress, which is a core factor in AUD. Thus, single housing is a potential confounding factor to be considered in voluntary ethanol drinking paradigms.

The few effects of ELS alone, compared to what expected, might be explained by allostatic mechanisms during development or confounding due to single housing in adulthood. ELS and ethanol drinking in adulthood exerted independent effects on hypothalamic and pituitary related genes, in a manner dependent on the control group used. As no regulatory effect of methylation in the targeted promoter regions was observed, further investigations is needed since correlative relationships between genes regulating DNA methylation and receptor genes in HPA axis were observed in rats exposed to ELS and ethanol drinking. The present study deepens understanding of the ELS mediated changes in genes related to the HPA axis and how they can affect the ELS-mediated propensity to ethanol drinking in adulthood. Moreover, it provides insight into the importance of the choice of the control group while studying the effects of ELS and indicates single housing as a potential confounding factor in ethanol drinking paradigms.

## Author Contributions

IN: study concept and design; LG: performing of animal experiment; AT: genetic analyses; AT, MA: epigenetic analyses; AT, EC, IN, KN: statistical analyses; IN, AT, EC, KN: interpretation of findings; AT, EC, IN: writing of the first draft; all authors: critical revision of the manuscript.

## Conflict of Interest Statement

The authors declare that the research was conducted in the absence of any commercial or financial relationships that could be construed as a potential conflict of interest.
