# Supplementary material for: HPA Axis Gene Expression and DNA Methylation Profiles in Rats Exposed to Early Life Stress, Adult Voluntary Ethanol Drinking and Single Housing
Source: Front Mol Neurosci. 2016 Jan 26;8:90. doi: 10.3389/fnmol.2015.00090 (PMC4726785; doi:10.3389/fnmol.2015.00090)
Supplement: Supplementary file 1 [file Data_Sheet_1.DOCX]

**SUPPLEMENTARY MATERIAL**

**HPA axis gene expression and DNA methylation profiles in rats exposed to early life stress, adult voluntary ethanol drinking and single housing**

Aniruddha Todkar ^1^, Linnea Granholm ^2^, Mujtaba Aljumah ^1^, Kent W , Nilsson ^3^, Erika Comasco ^1#*^, Ingrid Nylander ^2#*^

1 Department of Neuroscience, Uppsala University, Uppsala, Sweden

2 Department of Pharmaceutical Bioscience, Uppsala University, Uppsala, Sweden

1. Centre for clinical research, Västerås Central Hospital, Uppsala, Sweden

^#^ : equal contribution

**Supplementary Figure 1**

Gel electrophoresis proofs of primer specificity for the genes of interest and RNA integrity


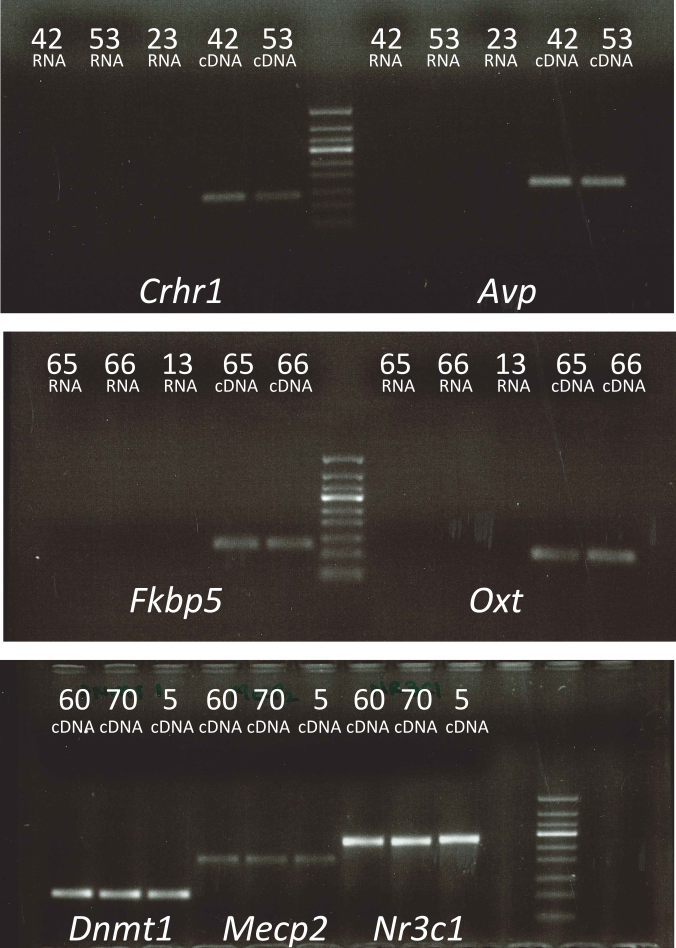


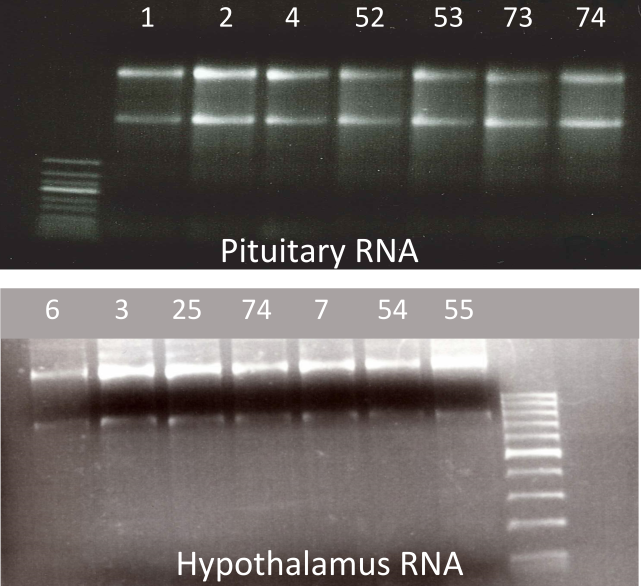


**DNA methylation assays**

**Choice of the region of interest:** Regarding the *Pomc* gene, the selected amplicon was a 268bp long region comprising a CpG island and many TFBSs. For the *Avp* gene, a CpG island was not present in the promoter region, and the selected amplicon consisted of a 142bp long sequence including a TFBS. For the *Crhr1* gene a 153bp long amplicon was targeted covering one CpG islands in the promoter region; while for the *Fkbp5* gene no information was available, therefore the amplicon was selected as a sequence of 168bp that is close to the TSS and which gave high probability of a successful assay. Target sequences are reported in Table S2 and figure S2.

DNA isolation: The DNA to assess the *Avp* and *Fkbp5* genes was extracted from the hypothalamus, while the DNA for the *Crhr1*, *Fkbp5*, and *Pomc* genes was extracted from the pituitary gland. Importantly, the DNA was isolated from the same cells clusters used for the messenger RNAs (mRNAs) isolation.

**Bisulfite conversion and pyrosequensing:** DNA underwent Bisulphite Conversion, with an efficiency of more than 99%, using EZ DNA methylation kit (ZymoResearch, Inc., CA), to convert un-methylated cytosine bases (C) into uracil bases (U). 0.2μM of each primer with one of the PCR primers being biotinylated was used to purify the final PCR product using sepharose beads. The PCR product was bound to Streptavidin Sepharose HP (Amersham Biosciences, Uppsala, Sweden), and the Sepharose beads containing the immobilized PCR product were purified, washed and denatured using 0.2M NaOH solution and rewashed using the Pyrosequencing Vacuum Prep Tool (Pyrosequencing, Qiagen) as recommended by the manufacturer. Pyrosequencing primer (0.5μM) was annealed to the purified single-stranded PCR product. 10μl of the PCR products were sequenced by Pyrosequencing PSQ96 HS System (Pyrosequencing, Qiagen), to detect the differentially methylated CpG sites, following the manufacturer’s instructions.

**Supplementary Figure 2**

Illustration of target regions and CpG sites for DNA methylation analyses
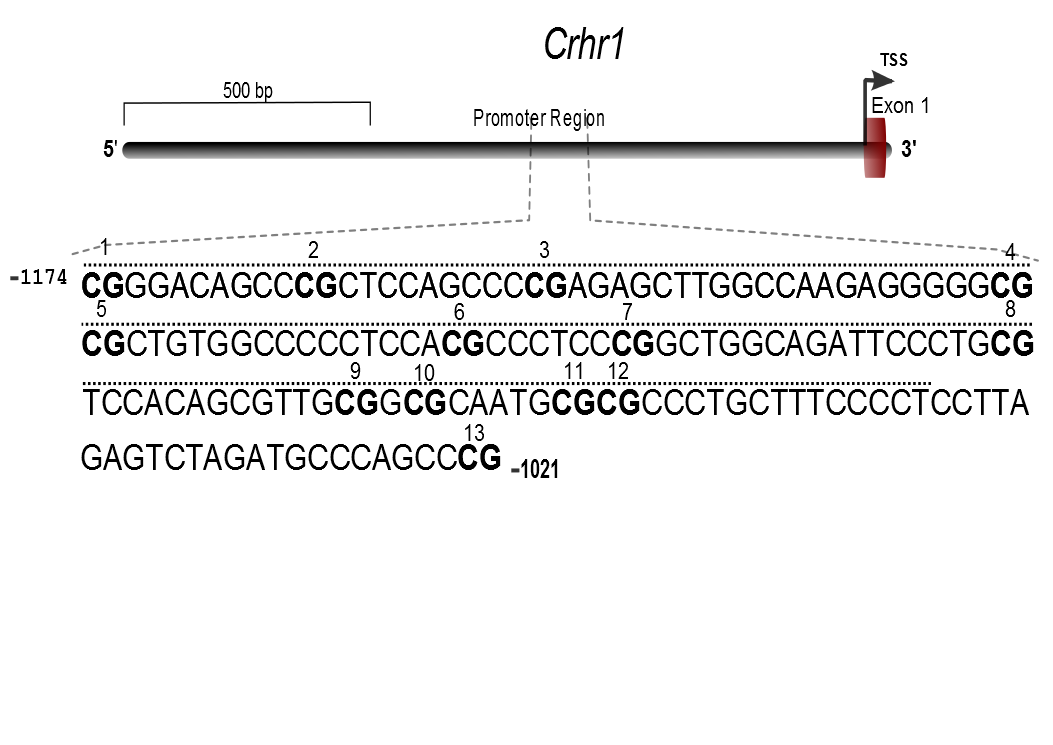


***Figure 2. Promoter region of the Crhr1 gene*** *DNA sequence showing a 153 bp fragment (-1174 to -1021 referring to TSS) and the location of the CpG sites (1–13)*


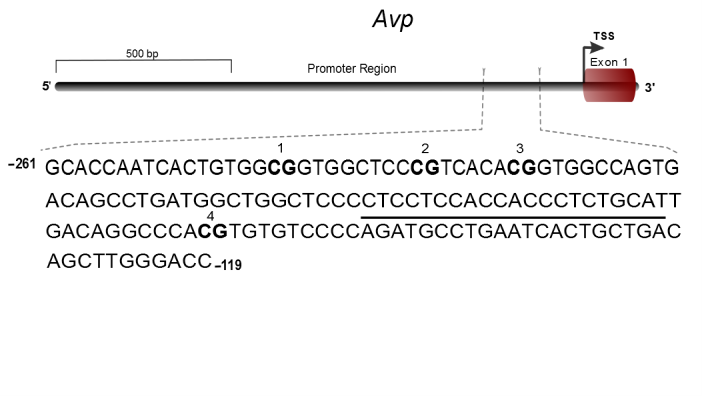


***Figure 1. Promoter region of the Avp gene****DNA sequence showing a 142 bp fragment (-261 to -119 referring to TSS) and the location of the CpG sites (1–4)*


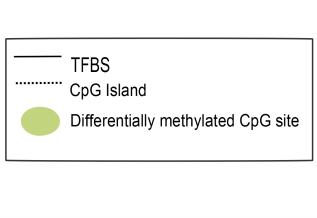


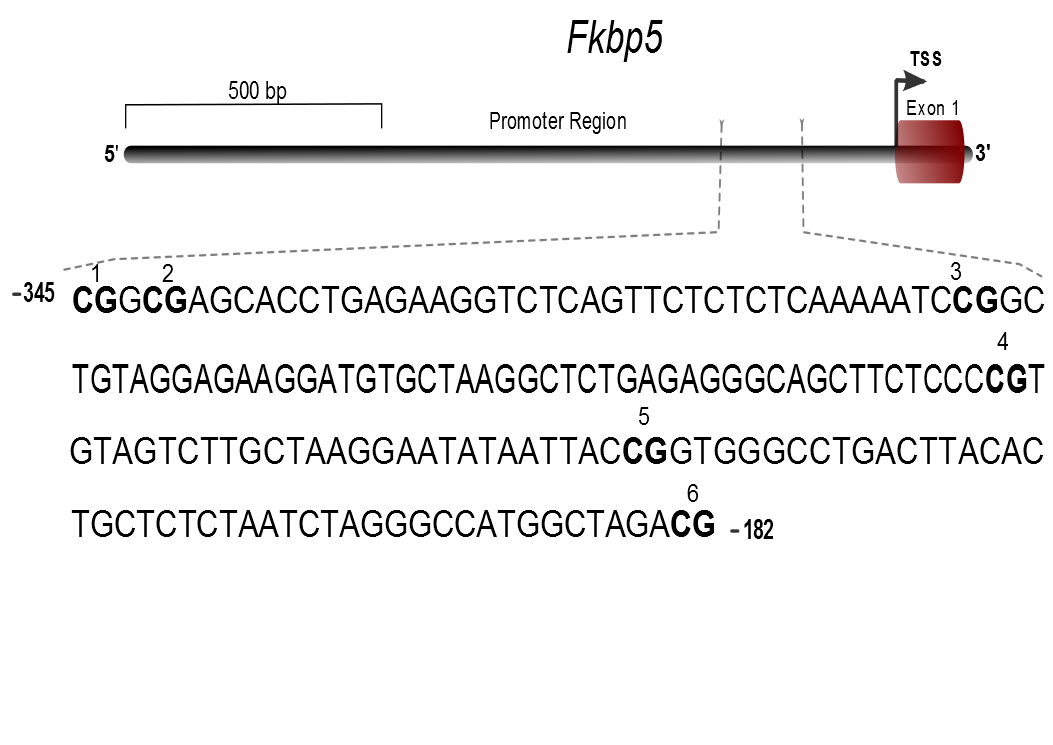


***Figure 4. Promoter region of the Fkbp5 gene****DNA sequence showing a 168 bp fragment (-345 to -182 referring to TSS) and the location of the CpG sites (1–6)*


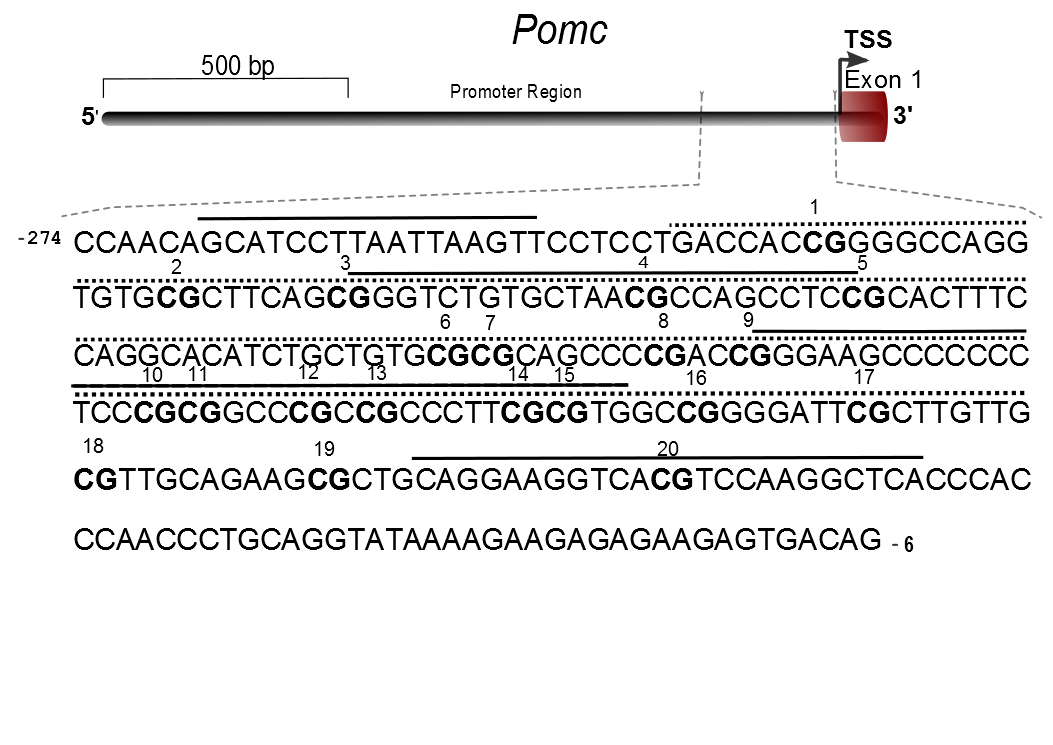


***Figure 3. Promoter region of the Pomc gene****DNA sequence showing a 268 bp fragment (-274 to -6 referring to TSS) and the location of the CpG sites (1–20)*

**Gene expression analyses**

**cDNA synthesis:** The manufacturer’s protocol was followed including a genomic DNA (gDNA) wipe-out reaction. The final cDNA synthesis reaction was carried out at 42^ᵒ^C for 35 minutes. The reaction was inactivated at 95^ᵒ^C for 5 minutes.

**Primer design and PCR cycling conditions:** Primers were designed using Primer 3 (http://frodo.wi.mit.edu/) and cross-checked using Primer Map (http://www.bioinformatics.org/sms2/primer_map.html) (table S1). For each sample, a 20µl reaction contained 1x [**iQ SYBR Green Supermix**](http://www.bio-rad.com/en-se/sku/170-8882-iq-sybr-green-supermix) (Biorad Sweden), 0.15µM of each primer, and 3 µl cDNA template. The PCR consisted of denaturation at 95^ᵒ^C for 3min followed by three step protocol with denaturation for 30 sec at 95^ᵒ^C, annealing for 30sec at the optimized temperature (Table S1), and elongation for 45sec at 72^0^C. Single plate read was taken at the end of each elongation step, and the melt curve was added after the end of PCR from 65^ᵒ^C to 95^ᵒ^C with 0.5^ᵒ^C/cycle increments. Each plate contained samples belonging to all experimental groups.

**Supplementary Table 1**

Primer pairs used to assess the gene expression with their respective annealing temperature

| **Gene** | **Gene name** | **Primers** | **T (^0^C)** |
| --- | --- | --- | --- |
| *Actb* | Actin, Beta | Forward: 5’ CACTGCCGCATCCTCTTCCT 3’  Reverse: 5’ AACCGCTCATTGCCGATAGTG 3’ | 60 |
| *Avp* | Arginine Vasopressin | Forward: 5’ ACCTCTGCCTGCTACTTCCA 3’ Reverse: 5’ CAG GAA GCA GCC CAG CTC 3’ | 61.4 |
| *Crhr1* | Corticotropin Releasing Hormone Receptor | Forward: 5’CATTGGCTGGGGTGTACCTT 3’ Reverse: 5’ATCATGGGGCCCTGGTAGAT 3’ | 61.4 |
| *Dnmt1* | DNA (Cytosine-5-)-Methyltransferase 1 | Forward: 5’CAATGAGGCACTGTCGTCT 3’ Reverse: 5’AAGTGACCGCGACTGCAATA 3’ | 63.3 |
| *Fkbp5* | FK506 Binding Protein 5 | Forward: 5’TGGTCTGACTCTCGTGTTTCTTG 3’ Reverse: 5’CGCAGGGTGTACGCCAAC 3’ | 63 |
| *Gapdh* | Glyceraldehyde-3-Phosphate Dehydrogenase | Forward: 5’ACATGCCGCCTGGAGAAACCT 3’  Reverse: 5’GCCCAGGATGCCCTTTAGTGG 3’ | 60 |
| *Mecp2* | Methyl CpG Binding Protein 2 | Forward: 5’CAGCTCCAACAGGATTCCATGGT 3’ Reverse: 5’TGATGTCTCTGCTTTGCCTGCCT 3’ | 63.3 |
| *Nr3c1* | Nuclear Receptor Subfamily 3, Group C, Member 1 (Glucocorticoid Receptor) | Forward: 5’ CTGAGGGGAGGAGCTACAGT 3’ Reverse: 5’ GGCCCAGTCATTCCCCATCA 3’ | 63.3 |
| *Oxt* | Oxytocin/Neurophysin I Prepropeptide | Forward: 5’ CTTGGCCTACTGGCTCTGAC 3’ Reverse: 5’ GTCCGCAGGGAAGACACTT 3’ | 62.3 |
| *Pomc* | Proopiomelanocortin | Forward: 5’ CAAGAGGGAGCTGGAAGGCGAGC 3’  Reverse: 5’ TCACTGGCCCTTCTTGTGC 3’ | 60 |
| *Rpl19* | Ribosomal Protein L19 | Forward: 5’ TCGCCAATGCCAACTCTCGTC 3’ Reverse: 5’ AGCCCGGGAATGGACAGTCAC 3’ | 62 |

**Supplementary table 2**

DNA methylation target sequences

| **Gene** | **ID (Ensemble)** |  | **Position (ref to TSS)** | **Number of CpG Sites** |
| --- | --- | --- | --- | --- |
| *Avp* | ENSRNOG00000021229 |  | -261 to -119 | 4 |
| *Crhr1* | ENSRNOG00000004900 |  | -1174 to -1021 | 13 |
| *Fkbp* | ENSRNOG00000022523 |  | -345 to -182 | 6 |
| *Pomc* | ENSRNOG00000012686 |  | -274 to -6 | 20 |
